# Tensor-valued diffusion MRI detects brain microstructure changes in HIV infected individuals with cognitive impairment

**DOI:** 10.21203/rs.3.rs-4482269/v1

**Published:** 2024-06-13

**Authors:** Md Nasir Uddin, Meera V. Singh, Abrar Faiyaz, Filip Szczepankiewicz, Markus Nilsson, Zachary D. Boodoo, Karli R. Sutton, Madalina E. Tivarus, Jianhui Zhong, Lu Wang, Xing Qiu, Miriam T. Weber, Giovanni Schifitto

**Affiliations:** University of Rochester; University of Rochester; University of Rochester; Lund University; Lund University; University of Rochester; University of Rochester; University of Rochester; University of Rochester; University of Rochester; University of Rochester; University of Rochester; University of Rochester

**Keywords:** b-tensor encoding, HIV, brain, neuroimaging, cognition, blood markers

## Abstract

Despite advancements, the prevalence of HIV-associated neurocognitive impairment remains at approximately 40%, attributed to factors like pre-cART (combination antiretroviral therapy) irreversible brain injury. People with HIV (PWH) treated with cART do not show significant neurocognitive changes over relatively short follow-up periods. However, quantitative neuroimaging may be able to detect ongoing subtle microstructural changes. This study aimed to investigate the sensitivity of tensor-valued diffusion encoding in detecting such changes in brain microstructural integrity in cART-treated PWH. Additionally, it explored relationships between these metrics, neurocognitive scores, and plasma levels of neurofilament light (NFL) chain and glial fibrillary acidic protein (GFAP). Using MRI at 3T, 24 PWH and 31 healthy controls underwent cross-sectional examination. The results revealed significant variations in b-tensor encoding metrics across white matter regions, with associations observed between these metrics, cognitive performance, and blood markers of neuronal and glial injury (NFL and GFAP). Moreover, a significant interaction between HIV status and imaging metrics was observed, particularly impacting total cognitive scores in both gray and white matter. These findings suggest that b-tensor encoding metrics offer heightened sensitivity in detecting subtle changes associated with axonal injury in HIV infection, underscoring their potential clinical relevance in understanding neurocognitive impairment in PWH.

## Introduction

Human immunodeficiency virus (HIV) infiltrates immune system cells and crosses the blood-brain barrier (BBB) shortly after seroconversion and leads to brain injury(1). This infiltration triggers a cascade of effects, including axonal disruption, myelin loss, astrogliosis, and to a lesser extent, (2). Approximately 50% of people with HIV (PWH) may experience mild cognitive impairment. These deficits can affect cognitive domains such as executive function, attention, fine motor skills, and information processing speed (2–4).

Despite the adoption of combination antiretroviral therapy (cART), chronic mild neuroinflammation is believed to be the primary reason for HIV-associated cognitive impairment. The key contributors to neuroinflammation are activated microglia and perivascular macrophages, with some involvement from astrocytes. An additional contribution is the transmigration to the central nervous system (CNS) of activated monocytes, which, after differentiation, increase the pool of perivascular macrophages (1, 5–8). As cART becomes more accessible, the understanding of brain abnormalities and cognitive deficits in HIV patients has become increasingly complex. Aging individuals receiving cART may develop comorbid medical conditions that independently lead to brain damage and cognitive changes. Furthermore, certain antiretroviral regimens have been associated with brain damage, complicating treatment strategies (9). Hence, there is an urgent need for advanced techniques to deepen our understanding of the pathogenesis of tissue changes in the brain due to HIV infection. Conventional approaches to evaluate neuronal injury involve serum or cerebrospinal fluid markers. However, these methods entail invasive procedures and lack the exceptional spatial resolution offered by magnetic resonance imaging (MRI). As a result, there is a growing interest in sensitive, reliable, readily accessible, and reproducible noninvasive imaging approaches for evaluating the brain injury. Although MRI doesn’t offer information at a cellular level, it enables the characterization of various biophysical tissue properties tightly coupled with neuroinflammatory processes.

Advanced MRI pulse sequences and post-processing methods provide novel quantitative measures reflective of brain injury (10–14). Utilizing micrometer-scale displacement of tissue water, diffusion MRI (dMRI) can noninvasively detect microstructural abnormalities in the brain (15–17). It provides excellent sensitivity to microstructural damage associated with HIV (18–21). However, conventional read-outs are significantly impacted by the dispersion of regional fiber orientations, such as crossing fibers, posing challenges in detecting regional pathology. This issue is not confined to standard diffusion tensor imaging (DTI) but extends to more advanced metrics when the effects of fiber orientation dispersion are not considered. For instance, the fractional anisotropy (FA) of white matter in DTI is closely linked to densely packed and myelinated axonal structures, as well as the presence of glial cells in disease (22, 23). However, interpreting the FA is challenging due to the blending of mesoscopic tissue features (e.g., fiber orientation dispersion and crossings) with microscopic features (e.g., axons, cells, and density). These complexities may result in FA changes misinterpreted as pathology (24). Given that 90% of white matter voxels involve crossing fibers, the imperfect alignment of axonal fibers makes it nearly impossible to separate tissue microstructural anisotropy from macrostructure using FA (25–28). For example, increased FA in Alzheimer’s disease was attributed to changes in fiber orientation dispersion rather than microscopic anisotropy alterations (29, 30).

Tensor-valued diffusion encoding is a new technique which employs diffusion encoding in multiple directions before image readout. While encoding in a single direction – as is done for DTI – yields linear tensor encoding (LTE), encoding in all directions with equal sensitivity yields spherical tensor encoding (STE). By contrasting LTE and STE, additional information about the tissue microstructure can be obtained, such as the separation of microscopic anisotropy and orientation dispersion (31). While a similar objective has been defined for many modelling methods using LTE, such methods are prone to bias due to modeling degeneracy (32). Unlike DTI, where the interpretation of FA relies on both microscopic features and the bulk tract orientation dispersion, b-tensor encoding separates these effects through diffusional variance decomposition (33–35). This approach enables the assessment of axonal integrity by measuring microscopic fractional anisotropy (μFA) as well as isotropic and anisotropic diffusional variance (MKi and MKa) at the sub-voxel level. Thus, b-tensor encoding measures may emerge as a sensitive biomarker for evaluating brain microstructure (both gray and white matter) in vivo (36, 37). To date, b-tensor encoding has been used to assess microstructural abnormalities in several diseases (34, 38–40).

In addition to advanced MRI, plasma levels of neurofilament light (NFL) chain and glial fibrillary acid protein (GFAP) serve as fluid biomarkers of brain injury. Although higher concentrations of NFL and GFAP are measured in cerebrospinal fluid, recent advances have increased the sensitivity of the assays making their assessment possible in peripheral blood samples.

The main goals of this work were to investigate whether b-tensor encoding metrics show better sensitivity to HIV infection compared to metrics from conventional DTI, and if they correlate with cognitive performance and blood markers.

## Methods and Materials

### Study Subjects

Twenty-four PWH (age = 55±10 years, male/female = 17/7) and 31 matched healthy controls (HC) (age = 55±15 years, male/female = 24/7) were enrolled from Rochester NY, and vicinity area. The Institutional Research Subjects Review Board (RSRB) at the University of Rochester thoroughly reviewed and approved the study. All participants provided written informed consent prior to enrollment and underwent clinical, laboratory, neurocognitive, and brain MRI examinations. All experiments were conducted in accordance with relevant guidelines and regulations. Detailed baseline demographics are presented in Table 1.

Our previous report (41), provides detailed descriptions of the inclusion and exclusion criteria as well as all study procedures. To briefly summarize, PWH meeting inclusion criteria had stable cART for a minimum of 3 months before screening and were aged ≥ 18. Exclusions encompassed individuals with symptomatic cardiovascular diseases (angina, myocardial infarction, stroke, or other peripheral atherosclerotic disease) and uncontrolled vascular risk factors such as diabetes mellitus and hypertension. Additionally, those with severe premorbid or comorbid psychiatric disorders (schizophrenia, bipolar disorder, active depression), brain infections other than HIV-1, space-occupying brain lesions, dementia from any cause, and metallic implants were excluded. The control population differed from PWH based on HIV status, level of education and race.

### Data Acquisition

#### Blood sample

Whole blood (~ 40 ml) was drawn into sterile, acid-citrate-dextrose (ACD) Vacutainer^®^ blood collection tubes. The plasma was then isolated and used for measuring specified markers. NFL and GFAP levels were measured using Single molecule array (Simoa^™^)(42) kits by Quanterix on a Simoa HD-1 analyzer (43–45).

### Neuropsychological assessments

Assessments of neurocognitive and functional performance were performed in all subjects. Before conducting analyses, Z-scores were computed for each cognitive domain, including a total Z-score for each participant. Study coordinators trained and supervised by an experienced neuropsychologist, administered all neuropsychological tests. The test battery covered diverse cognitive domains, such as Attention/Working Memory (CalCAP CRT 4; CRT 14), Speed of Information Processing (Stroop Color Naming, Digit Symbol Modalities Test), Executive Function (Trail Making Test B, Stroop Interference Task), Language (letter and category fluency), Learning (Rey Auditory Verbal Learning Test Trials 1–5, Rey Complex Figure Test Immediate Recall), Memory (RAVLT Trial 7, RCFT Delayed Recall), and Motor Skill (Grooved Pegboard). Assessment of premorbid intellectual functioning and English language fluency was limited to the baseline, utilizing the Wide Range Achievement Test (WRAT) 4-Reading subtest.

### Magnetic Resonance Imaging

MRI was performed on a 3T whole-body scanner (MAGNETOM Prisma Fit, Siemens, Erlangen, Germany, software version VE11c) equipped with a 64-channel head coil. The maximum gradient strength is 80 mT/m with a slew rate of 200 mT/m/s.

Anatomical Imaging: The T1-weighted (T1w) images were acquired using a 3D magnetization prepared rapid acquisition gradient-echo (MPRAGE) sequence with inversion time (TI) = 926 ms, repetition time (TR) = 1840 ms; echo time (TE) = 2.45 ms; flip angle = 8°; Field of View (FOV) = 256×256; GRAPPA = 2; voxel size = 1.0×1.0×1.0 mm^3^. 3D T2-weighted FLAIR images were acquired with scan parameters: TI = 1,800 ms, TR = 5,000 ms, TE = 215 ms, voxel size = 1.0×1.0×1.0 mm^3^.

Tensor-valued or b-tensor encoding: We employed a prototype pulse sequence that accommodates free waveform encoding sequence (FWF version 1.13s), available at https://github.com/filip-szczepankiewicz/fwf_seq_resources the diffusion-weighted single-shot spin echo sequence (33, 46) with encoding waveforms that were compensated for concomitant gradient effects. The imaging protocol involved acquiring 43 images with 43 linear b-tensor encoding (LTE) and 37 with spherical tensor encoding (STE), spread across multiple b-values (b = 0, 100, 700, 1400, and 2000 ms/m^2^). The imaging parameters included TR = 4100 ms, TE = 91 ms, FOV = 224×224 mm^2^, partial-Fourier = 6/8, GRAPPA factor = 2, echo spacing (ESP) = 0.6 ms, and voxel size = 2×2×4 mm^3^. The imaging stack was axial, with phase encoding along the anterior-posterior direction.

Diffusion Tensor Imaging (DTI): Employing a single-shot spin-echo, echo-planar imaging (SE-EPI) pulse sequence and the simultaneous Multi-Slice (SMS) acquisition technique, diffusion weighted imaging (DWI) was conducted with the following parameters: TR = 3000ms, TE = 67ms, b = 0, 1000, 2000 s/mm^2^, 64 directions/shell with 6 b = 0, GRAPPA = 2, multiband factor = 3, and voxel size = 1.25×1.25×1.25 mm^3^. To correct for distortions, images with opposite polarity (i.e., AP and PA directions) were acquired.

### Data Analysis

Image analyses were performed using a combination of image processing tools, including FSL (https://fsl.fmrib.ox.ac.uk/fsl/fslwiki/ (47), ANTs (http://stnava.github.io/ANTs/) (48), and MATLAB (*version 2021b*).

All MR images underwent thorough inspection for artifacts, including motion, geometric distortion, and signal dropout. T1w images underwent structural segmentation using the anatomical processing script (fsl_anat) from FMRIB’s Software Library (FSL) (49). The processing pipeline involved image reorientation and cropping, radio-frequency bias-field correction, linear and nonlinear registration to MNI 2mm standard space through FLIRT and FNIRT, brain extraction via BET(50), tissue segmentation using FAST, and subcortical structure segmentation employing the FIRST algorithm. Lesion segmentation was carried out using volBrain, an automated online MRI brain volumetry system(51), based on T1w and FLAIR images.

Tensor-valued or b-tensor encoding: NIfTI images were prepared for compatibility with the multidimensional diffusion MRI framework (52) available at https://github.com/markus-nilsson/md-dmri. Briefly, the diffusion-weighted images from each participant underwent a three-step processing approach: 1) Correction for eddy current-induced distortion and inter-volume subject motion was achieved by registering the images to an extrapolated reference (53) using ElastiX (54). The use of extrapolation-based references is crucial for accurate registration of high b-value images. 2) Smoothing of the images was carried out using a 3D Gaussian kernel with a standard deviation of 0.4 voxels. 3) Voxel-by-voxel normalized anisotropic, isotropic, and total diffusional variance (MKa, MKi, MKt), as well as microscopic anisotropy (μFA), were obtained through linear least squares fitting of the log signal while correcting for heteroscedasticity.

DTI: The DWI were corrected for eddy current-induced distortion, inter-volume subject motion, and susceptibility-induced distortion using “topup” and “eddy” tools in FSL (55, 56). DTI metrics such as FA and MD were computed by using DTIFIT in FSL(49).

#### ROI analysis:

For pre-specified regions of interest (ROIs), we calculated average ROI values for all MRI metrics (DTI – FA, MD; b-tensor encoding - μFA, MKi, MKa, MKt). We used Harvard-Oxford cortical and subcortical, and the Johns Hopkins University WM (JHU-WM) atlases available in FSL in standard MNI152–2 mm space for ROI extraction. Prior to this, all MRI metrics were registered to the high-resolution T1w images of the same individual using a 12-DOF linear registration (FLIRT tool in FSL). Then, individuals’ T1w images were spatially normalized to the MNI-T1–152 standard template using nonlinear registration (using ANTs) (48, 57). The transformation matrix and the warping field from these two steps were applied to DTI, and b-tensor encoding metrics. We then extracted mean values from the MRI metrics for the followings: global white matter (GWM), cortical gray matter (CGM), subcortical gray matter (SGM) using the corresponding masks as ROIs, and four white matter tracts encompassing coherent, crossing, and fanning fibers: Genu of Corpus Callosum (GCC), Anterior Corona Radiata (ACR), Forceps Minor (FMin), Superior Fronto-Occipital Fasciculus (SFOF).

#### Statistical Analyses

Statistical analyses were performed in Python (version 3.7.4) and MATLAB (version R2022b). An unpaired t-test was used to compare the differences in ROIs between the two cohorts. Spearman correlation analyses were performed to find the associations between imaging metrics and blood markers and cognitive scores after controlling for age. A p-value of < 0.05 was considered statistically significant for a single hypothesis testing problem. For inferential problems that involved multiple hypotheses, the Benjamini–Hochberg multiple testing procedure was used to control the false discovery rate (FDR) at the < 0.05 level (58).

## Results

### Participant Characteristics

Detailed information about demographic, clinical, neurocognitive and MRI data of the study participants are presented in Table 1. The Welch’s Two Sample t-test did not reveal any statistically significant age difference between the HC and PWH (p = 0.947). Nonetheless, we incorporated age as a covariate in all multivariate regression analyses to mitigate any lingering confounding effects. Furthermore, in comparison to PWH, those who were HC exhibited significantly higher education levels and were to a higher degree Caucasians (p < 0.001).

#### Group comparisons of MRI metrics:

**Figure 1:**
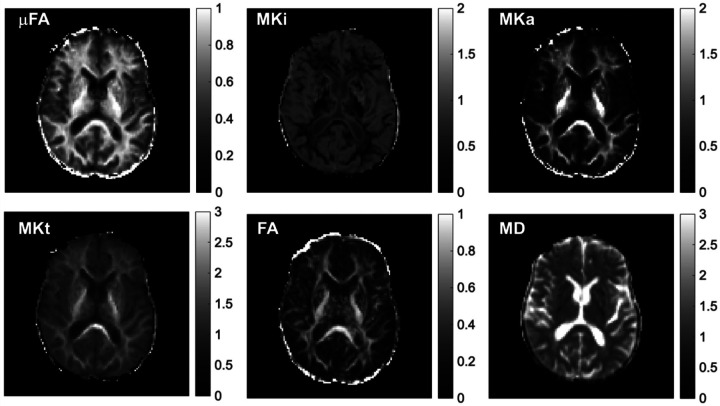
Example tensor-valued diffusion encoding maps (μFA, MKi, MKa, MKt,) as well as DTI maps (FA and MD) are presented from a 62-year-old individual with HIV. Intensity scale is also shown.

**Figure 2:**
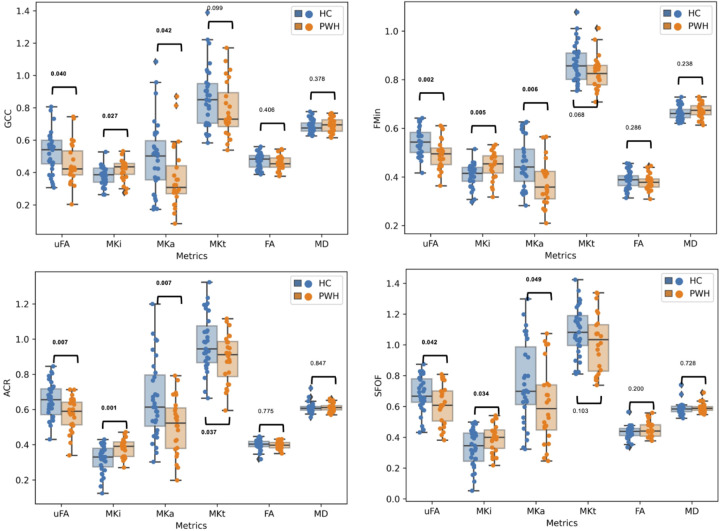
Comparison of dMRI metrics. B-tensor encoding metrics show significant differences between PWH and HC cohorts in white matter regions with coherent, crossing, and fanning fibers. GCC: Genu of corpus callosum; ACR: anterior corona radiata; FMin: Forceps Minor; SFOF: superior fronto-occipital fasciculus; Significant p-values are shown as bold; MD values are expressed as x10^−3^.

Fig. 1 represents mean voxel-by-voxel b-tensor encoding and DTI maps from a PWH subject. Our analysis revealed significant differences in b-tensor metrics in several white matter ROIs, while no significant findings were observed for DTI metrics (Table 2). In Fig. 2, we illustrate the comparisons between PWH and HC cohorts across various white matter tracts, encompassing coherent, crossing, and fanning fibers. Notably, we observed a significant decrease in μFA (p = 0.042 for GCC, p = 0.002 for FMin, p = 0.007 for ACR, p = 0.042 for SFOF) and MKa (p = 0.042 for GCC, p = 0.006 for FMin, p = 0.007 for ACR, p = 0.049 for SFOF), along with a significant increase in MKi (p = 0.027 for SCC, p = 0.005 for FMin, p = 0.001 for ACR, p = 0.034 for SFOF) among PWH. Although FA exhibited a decrease in PWH, it did not reach statistical significance. We also found increased MD in PWH cohort but not significant (not shown). The trend of changes in DTI metrics is consistent with previous works (19, 59–63). For example, several previous studies reported that PWH had a decreased FA in several brain regions, including genu and splenium of corpus callosum (GCC, SCC), and SFOF (19, 59–63). Although PWH had a decreased μFA and MKa and increased MKi compared to healthy controls in global ROIs i.e., for GWM, CGM and SGM, none of the metrics exhibited significant difference.

### Group comparisons of cognitive performance and blood markers

Welch’s two group t-test showed the total cognitive score was lower in the PWH cohort compared to the HC cohort (t = 2.22, p = 0.030). However, while the average concentrations of NFL and GFAP were slightly elevated in the PWH cohort compared to the HC cohort, these differences did not reach statistical significance.

### Relationship between cognitive scores and b-tensor metrics

**Figure 3:**
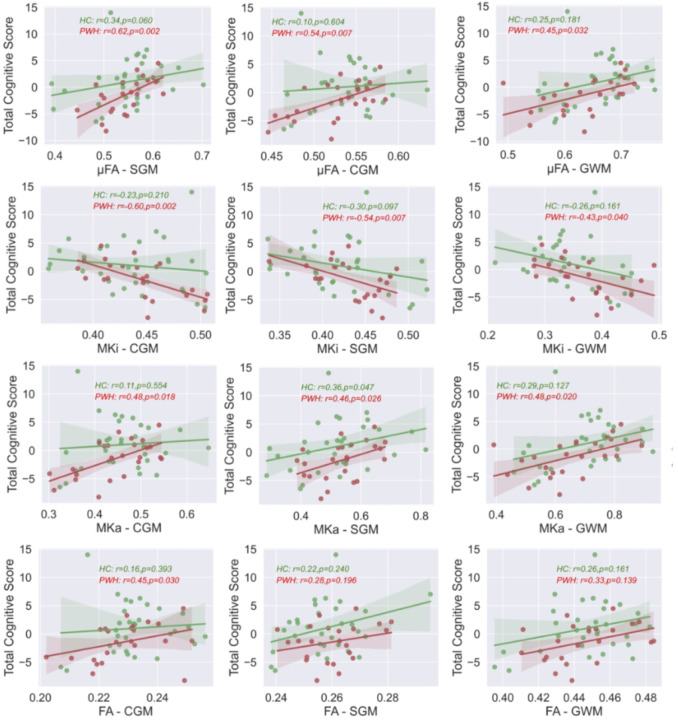
Relationships between Total cognitive scores and b-tensor encoding metrics in cortical gram matter (CGM), subcortical gray matter (SGM), global white matter. PWH: People with HIV, HC: Healthy controls.

We investigated the correlation between total cognitive z-scores and b-tensor encoding-based μFA, MKi, and MKa in global white matter (WM), subcortical gray matter (SGM), and cortical gray matter (CGM), among both PWH and HC individuals (see Fig. 3). Significant relationships were observed between total cognitive z-scores and b-tensor metrics in the PWH cohort, while no statistical significance was found for HC subjects except for MKa in SGM. Additionally, no significant associations were found for FA, except for CGM in PWH. Correlations between total cognitive scores and b-tensor encoding metrics in four white matter tracts, which involve coherent, crossing, and fanning fibers (GCC, ACR, FMin, and SFOF), are presented in **Supplementary Fig. 1**. Similar trends of changes were identified within those ROIs.

Further, we conducted correlation analyses between cognitive domain scores (i.e., Attention/Working Memory, Speed of Information Processing, Executive Function, Language, Learning, Memory, and Motor Skills) and b-tensor metrics, as well as DTI metrics, specifically μFA and FA for global ROIs (see **Supplementary Table 1**). Our findings suggest that executive function, attention, and motor skills display increased sensitivity to microstructural tissue changes measured by b-tensor encoding compared to DTI metrics. The trend of correlations aligns with previous studies involving DTI-derived FA.

We also performed two-way ANOVA to measure the effects of HIV status, MRI metrics of five ROIs (such as GWM, CGM and SGM, GCC and ACR) and their interactions on cognitive scores. Table 3 shows the representative results for total cognitive scores while **Supplementary Tables 2**–**4** are for sub-domains (such as executive function, attention, and motor functions).

#### Relationship between blood markers and b-tensor metrics:

**Figure 4:**
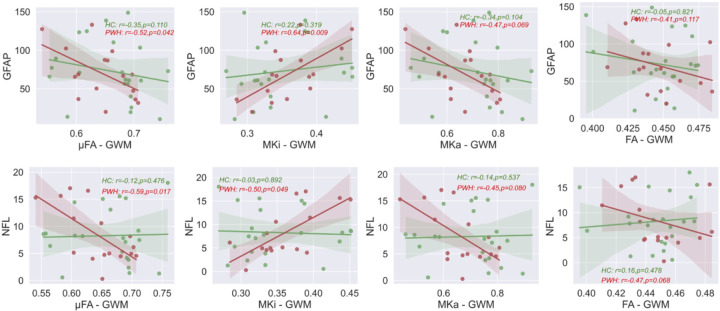
Relationship between blood markers (NFL and GFAP) and b-tensor metrics. Scatterplots of blood markers and b-tensor measures (μFA, MKi and MKa) in global white matter (WM) for each cohort. Regression lines are drawn with 95% confidence intervals. Spearman correlation coefficients and corresponding p-values are displayed within each plot for each cohort.

Fig. 4 illustrates the associations between average neurofilament light chain (NFL) concentrations, as well as GFAP with b-tensor and DTI metrics. NFL concentrations showed a negative correlation with μFA and MKa while being positively correlated in WM, and significance is mostly found in PWH subjects (p < 0.05). GFAP also shows similar trends. We did not find any significant interactions between HIV status and MRI metrics with blood markers.

## Discussion

This is the first study to apply diffusion MRI with b-tensor encoding in the context of HIV-associated neuropathology to better understand the underlying brain tissue microstructure and to investigate the association between b-tensor metrics and cognitive performance and blood markers of brain injury. Tensor-valued diffusion encoding proves valuable in unraveling orientation dispersion and sub-voxel anisotropy, surpassing the capabilities of conventional diffusion techniques like DTI, as it increases the amount of microstructure information encoded into the diffusion-weighted images(32). Our hypothesis posits that axonal injury would associate with elevated plasma levels of NFL and GFAP and lower cognitive performance in PWH. Our findings reveal that a) b-tensor encoding metrics (μFA, MKa, MKi, MKt) demonstrate stronger sensitivity to microstructural changes in the brain attributed to HIV infection than DTI metrics (FA and MD); b) b-tensor encoding metrics are significantly associated with cognitive scores in PWH but not with FA and MD; and c) b-tensor encoding metrics in white matter are significantly associated with blood markers such as GFAP and NFL in PWH.

In alignment with observations in other neuroinflammatory and neurodegenerative disorders (24, 64–66), PWH exhibit a reduction in anisotropy-related metrics (FA, μFA, MKa) and an elevation in diffusivity-related metrics (MD, MKi) compared to their healthy counterparts. This indicates a widespread loss of tissue microstructural integrity and possible edema. The mean values for diffusion metrics (both DTI and b-tensor encoding) align with previous studies involving both healthy and diseased subjects (67).

Given that both FA and μFA serve as indices of diffusion anisotropy, their comparability is noteworthy. Mean FA values typically ranged from 0.39 to 0.48 in white matter and 0.22 to 0.28 in gray matter, consistently lower than mean μFA values, which fell in the range of 0.49 to 0.76 in white matter and 0.44 to 0.64 in gray matter, respectively. This discrepancy is likely attributed to the presence of crossing and fanning fibers in brain tissues, which attenuate FA measurements without affecting μFA. The results suggest that μFA, along with other b-tensor encoding metrics, serves as a more sensitive and specific measure for detecting microstructural changes compared to FA.

Our findings indicate significant reductions by 10–15% in μFA values in various white matter regions, specifically in coherent (genu of corpus callosum), crossing (SFOF), and fanning (ACR) fibers, while FA showed up to 3% non-significant changes, in PWH compared to healthy controls. This implies that microstructural changes, as measured by μFA, are predominantly due to the loss of local anisotropy rather than disruption of white matter fiber coherence in the HIV cohort. Since μFA is proposed as a measure of axonal integrity rather than myelin (23, 68), this decrease in μFA suggests widespread axonal damage resulting from HIV infection. Notably, FA values were observed to be higher in some ROIs in individuals with HIV compared to healthy controls. For instance, FA increased by 2% in the SFOF, while μFA exhibited a 10% decrease compared to controls. One plausible explanation is that axonal degradation within a single bundle in a crossing fiber region, such as the SFOF, may diminish the regional dispersion of fiber orientation, consequently leading to an increase in regional FA. Furthermore, we observed a 17–24% decrease in MKa and up to a 4–10% decrease in MKt, along with a 10–21% increase in MKi, in white matter regions in PWH compared to controls. This is noteworthy, as conventional dMRI without b-tensor encoding cannot separate MKi and MKa, as it can only detect MKt, which is the sum of the two. By using b-tensor encoding to dissociate the two, larger differences between the groups were found.

The cognitive performance, as measured by the total cognitive Z-score, demonstrated a stronger correlation with b-tensor encoding metrics compared to DTI metrics in PWH, underscoring the sensitivity of b-tensor encoding metrics. Significantly, the interaction between b-tensor metrics and HIV status was observed for the b-tensor encoding-based anisotropy metrics (i.e., μFA and MKa in crossing and fanning fiber regions). This suggests that the cognitive effects in the HIV cohort are primarily linked to the loss of local anisotropy, impacting cognitive performance. Moreover, cognitive domain scores, particularly in executive function, attention, and motor functions, exhibited robust associations with anisotropy metrics, specifically μFA, compared to FA. However, there were no significant interactions between the imaging metrics and cognitive domain scores. The observed trend in correlations aligns with previous studies involving DTI-derived FA. Decreased FA has also been noted in various white matter regions, correlating with decreased memory and executive function in PWH exhibiting HIV-associated neurocognitive disorders, particularly in studies with larger sample size (63, 69, 70).

The presence of Neurofilament light chain (NfL) in plasma has emerged as blood marker indicative of neuroaxonal degradation (71). NfL is released into the brain’s extracellular space (ECS) following axonal injury and subsequently detected in the cerebrospinal fluid (CSF) and blood (71, 72). Elevated NfL levels consistently manifest in various neurological and neurodegenerative disorders, including HIV infection (72–75). Furthermore, activated glial cells are recognized for releasing microparticles expressing Glial Fibrillary Acidic Protein (GFAP) into circulation during brain injury (76–80), cognitive impairment (81), and viral infections such as HIV infection(82, 83). This study unveils a notably stronger association between b-tensor encoding metrics and both NFL and GFAP in white matter, compared to DTI metrics in individuals with HIV. This finding suggests enhanced sensitivity in detecting relevant correlations.

However, it is essential to acknowledge several limitations within this study. Firstly, despite the careful age matching between PWH and healthy controls, there exists an imbalance in the proportion of male and female participants. This discrepancy could introduce gender-related confounding factors. Despite concerted efforts to include female participants, the representation remains at a minimum of 25% in each cohort. Nevertheless, the proportion of males and females in both cohorts are not significantly different. However, the proportion of Caucasians and African Americans was significantly imbalanced. Moreover, due to a lack of SMS in the early implementation of the FWF sequence the collection of b-tensor encoding metrics encountered limitations in resolution compared to DTI measures (2×2×4 mm^3^ vs. 1.5×1.5×1.5 mm^3^). This discrepancy was a result of time constraints during data acquisition. These limitations should be considered when interpreting the results and may warrant further investigation in future studies with larger and more diverse cohorts.

## Conclusion

In this study, we investigated the effectiveness of tensor-valued diffusion encoding and associated analysis in delineating tissue microstructural degradation in people living with HIV. Our findings indicate that metrics based on b-tensor encoding demonstrate greater sensitivity in quantifying subtle changes associated with HIV infection. Moreover, we demonstrated a significant correlation between b-tensor encoding metrics, cognitive scores, and plasma levels of NFL and GFAP in PWH. Therefore, the utilization of b-tensor encoding offers a more comprehensive and clinically relevant insight into abnormalities in brain tissue microstructure related to HIV infection compared to the conventional DTI approach.

## Figures and Tables

**Table 1:** Subject Demographics.

Characteristics		PWH (n = 24)	HC (n = 31)	p-value
**Age, mean (SE)**		55.04 (1.96)	54.81 (2.71)	0.947
**Sex, n (%)**				0.443
	Female	7 (29.17%)	7 (25.81%)	
	Male	17 (70.83%)	24 (74.19%)	
**Ethnicity, n (%)**				0.847
	Hispanic or Latino	0	2 (3.8%)	
	Not Hispanic or Latino	23 (95.83%)	29 (96.2%)	
	Other	1 (4.17%)	0	
**Race, n (%)**				**<0.001**
	Caucasian	16 (66.67%)	27 (87.09%)	
	Black AA	6 (25%)	4 (12.90%)	
	Other	1 (4.16%)	0 (0%)	
	Missing	1 (4.16%)	0 (0%)	
**Education, n (%)**				**<0.001**
	≤ 12 Years	4 (16.67%)	1 (3.23%)	
	> 12 Years	20 (83.33%)	30 (96.77%)	
**Cognitive Z Scores, mean (SE)**				
	Total Cognitive Score	−1.39 (0.67)	1.00 (0.79)	**0.031**
**Blood Markers, mean (SE)** (PWH: N = 17, HC: N = 22),				
	NFL	8.82 (1.32)	8.27 (1.08)	0.749
	GFAP	74.57 (8.85)	72.91 (8.39)	0.893

Note: Continuous variables are summarized as Mean (Standard Error), categorical variables are summarized as frequency (percentages); Significant p-values are shown as bold. NFL: Neurofilament light chain; GFAP: glial fibrillary acidic protein.

**Table 2:** Diffusion MRI metrics for HIV and healthy controls in brain tissues Bold font indicates correlations that were statistically significant after FDR correction.

	Cohort	HC	PWH	% difference between cohorts	t-value	p-value
**GCC**	μFA	0.53 (0.123)	0.45 (0.034)	−15.09%	2.11	0.040
	MKa	0.49 (0.23)	0.37 (0.225)	−24.49%	2.08	0.042
	MKi	0.38 (0.059)	0.42 (0.034)	10.53%	−2.27	0.027
	MKt	0.87 (0.188)	0.78 (0.173)	−10.34%	1.84	0.099
	FA	0.47 (0.047)	0.46 (0.045)	−2.13%	0.83	0.406
	MD	0.68 (0.039)	0.69 (0.046)	1.47%	−0.88	0.378
**ACR**	μFA	0.65 (0.101)	0.57 (0.097)	−**12.31%**	**2.81**	**0.007**
	MKa	0.66 (0.222)	0.51 (0.161)	−**22.73%**	**2.81**	**0.007**
	MKi	0.32 (0.078)	0.38 (0.051)	**18.75%**	−**3.40**	**0.001**
	MKt	0.98 (0.161)	0.89 (0.140)	−9.18%	2.21	0.037
	FA	0.40 (0.028)	0.39 (0.023)	−0.52%	0.28	0.775
	MD	0.61 (0.029)	0.61 (0.024)	0.24%	−0.19	0.847
**FMin**	μFA	0.55 (0.057)	0.49 (0.063)	−**10.91%**	**3.28**	**0.002**
	MKa	0.46 (0.101)	0.38 (0.099)	−**17.39%**	**2.86**	**0.006**
	MKi	0.41 (0.047)	0.45 (0.058)	**9.76%**	−**2.91**	**0.005**
	MKt	0.87 (0.791)	0.83 (0.069)	−4.60%	0.28	0.068
	FA	0.39 (0.037)	0.38 (0.033)	−2.56%	1.08	0.286
	MD	0.66 (0.029)	0.67 (0.031)	1.52%	−1.19	0.238
**SFOF**	μFA	0.68 (0.116)	0.61 (0.129)	−10.29%	2.08	0.042
	MKa	0.76 (0.264)	0.62 (0.253)	−18.42%	2.01	0.049
	MKi	0.33 (0.121)	0.40 (0.092)	21.21%	−2.18	0.034
	MKt	1.09 (0.159)	1.01 (0.188)	−7.33%	1.67	0.103
	FA	0.44 (0.043)	0.45 (0.053)	2.28%	−1.29	0.200
	MD	0.58 (0.039)	0.59 (0.031)	0.60%	−0.35	0.728

**Table 3:** Two-Way ANOVA to measure the effects of imaging metrics, HIV status, and their interactions on total cognitive scores.

		μFA				MKi				MKa			
B-tensor encoding metrics vs. Total Cognitive Z score		Estimate	Std. Error	t value	Pr(>|t|)	Estimate	Std. Error	t value	Pr(>|t|)	Estimate	Std. Error	t value	Pr(>|t|)
**Global White Matter** **(GWM)**	Intercept	−14.15	7.92	−1.79	0.080	8.92	4.03	2.21	**0.031**	−7.12	3.87	−1.84	0.072
	GWM	22.88	11.92	1.92	0.060	−22.87	11.47	−1.99	0.052	11.54	5.42	2.13	**0.038**
	HIV Status	−3.62	11.25	−0.32	0.749	−0.48	6.37	−0.08	0.941	−2.93	5.55	−0.53	0.600
	HIV-GWM interaction	2.88	17.28	0.17	0.868	−3.87	17.53	−0.22	0.826	1.71	8.06	0.21	0.833
**Cortical Gray Matter** **(CGM)**	Intercept	−4.74	9.33	−0.50	0.613	7.68	7.05	1.09	0.281	−1.25	4.31	−0.29	0.772
	CGM	10.65	17.24	0.61	0.539	−15.24	16.03	−0.95	0.346	4.94	9.30	0.53	0.598
	HIV Status	−22.63	14.13	−1.60	0.115	16.25	11.91	1.37	0.178	−12.50	6.63	−1.89	0.065
	HIV-CGM Interaction	38.62	26.46	1.45	0.151	−41.82	26.86	−1.56	0.126	22.73	14.51	1.57	0.123
**Subcortical Gray Matter** **(SGM)**	Intercept	−7.93	5.51	−1.43	0.156	11.57	5.65	2.05	**0.046**	−4.57	2.91	−1.57	0.122
	SGM	16.29	9.98	1.63	0.109	−25.16	13.36	−1.88	0.065	10.75	5.45	1.97	0.054
	HIV Status	−17.15	10.98	−1.56	0.125	6.43	10.93	0.59	0.559	−5.54	5.56	−1.00	0.324
	HIV-SGM Interaction	27.18	20.03	1.35	0.181	−19.67	25.35	−0.78	0.441	5.48	10.26	0.53	0.596
**Genu of Corpus Callosum** **(GCC)**	Intercept	6.04	3.08	1.964	0.055	−7.89	4.58	−1.72	0.091	3.23	1.70	1.90	0.063
	GCC	−9.56	5.68	−1.682	0.099	23.31	11.87	1.96	0.055	−4.56	3.17	−1.44	0.156
	HIV Status	−10.10	4.28	−2.361	**0.022**	6.71	7.10	0.95	0.349	−6.16	2.43	−2.54	**0.014**
	HIV-GCC Interaction	15.44	8.49	1.819	0.075	−23.83	17.47	−1.36	0.179	8.76	5.25	1.67	0.101
**Anterior Corona Radiata** **(ACR)**	Intercept	3.58	4.55	0.786	0.436	1.41	3.05	0.46	0.645	1.87	2.18	0.86	0.394
	ACR	−3.96	6.92	−0.572	0.570	−1.29	9.26	−0.14	0.890	−1.32	3.14	−0.42	0.675
	HIV Status	−16.36	6.61	−2.474	**0.017**	5.86	6.95	0.84	0.404	−9.45	3.42	−2.77	**0.008**
	HIV-ACR Interaction	23.79	10.76	2.211	**0.032**	−21.36	18.67	−1.14	0.258	13.48	5.86	2.30	**0.025**

## Data Availability

Anonymized data will be made available on reasonable request, pending appropriate institutional review board approvals.
